# The Role of Gut Microbiota in Chronic Itch-Evoked Novel Object Recognition-Related Cognitive Dysfunction in Mice

**DOI:** 10.3389/fmed.2021.616489

**Published:** 2021-02-05

**Authors:** Yujuan Li, Wencui Zhang, Tainning Sun, Baowen Liu, Anne Manyande, Weiguo Xu, Hong-Bing Xiang

**Affiliations:** ^1^Department of Anesthesiology, Tongji Hospital, Tongji Medical College, Huazhong University of Science and Technology, Wuhan, China; ^2^School of Human and Social Sciences, University of West London, London, United Kingdom; ^3^Department of Orthopedics, Tongji Hospital of Tongji Medical College, Huazhong University of Science and Technology, Wuhan, China

**Keywords:** chronic itch, gut microbiota, novel object recognition, cognitive dysfunction, metabolites

## Abstract

The high incidence of patients with chronic itch highlights the importance of fundamental research. Recent advances in the interface of gut microbiota have shed new light into exploring this phenomenon. However, it is unknown whether gut microbiota plays a role in chronic itch in rodents with or without cognitive dysfunction. In this study, the role of gut microbiota in diphenylcyclopropenone (DCP)-evoked chronic itch was investigated in mice and hierarchical cluster analysis of novel object recognition test (ORT) results were used to classify DCP-evoked itch model in mice with or without cognitive dysfunction (CD)-like phenotype and 16S ribosomal RNA (rRNA) gene sequencing was used to compare gut bacterial composition between CD (Susceptible) and Non-CD phenotypes (Unsusceptible) in chronic itch mice. Results showed that the microbiota composition was significantly altered by DCP-evoked chronic itch and chronic itch induced novel object recognition-related CD. However, abnormal gut microbiota composition induced by chronic itch may not be correlated with novel object recognition-related CD.

## Introduction

Everyone has experienced symptoms of pain and itching stress. According to present studies, pain and itching share close associations in sensory perceptions and neural pathways (1–3). Similar to pain, itching is classified into two forms. Acute itching is easily reduced by scratching or anti-allergic drugs whereas chronic repetitive itching remains a challenge to clinic cure (4–7). It has been reported that the itch-scratch cycle plays an important role in the maintenance of the chronic itch (8–11). Furthermore, the itch-scratch cycle is centered around three key points of itch neurobiology and skin immunology (12): the epithelial barrier, the peripheral nervous system and the immune system dysfunction (13, 14). While well-known clinically, its mechanisms have historically lacked in-depth understanding, possibly due to the complexity of the chronic itch.

Several studies have reported that gut microbiota and their dependent metabolites are associated with the development of chronic diseases (15, 16). Cani et al. (17) demonstrated that changes in gut microbiota control metabolic endotoxemia-induced inflammation in high-fat diet-induced obesity and diabetes in mice. Ke et al. (18) observed a change in gut flora-dependent metabolite Trimethylamine-N-oxide (TMAO) during the aging process and the effects of TMAO on chronic cardiovascular diseases, and reported that TMAO accelerated endothelial cell senescence and vascular aging and increased oxidative stress through the activation of the p53/p21/Rb pathway. Thus, we speculate that gut microbiota may affect the development of chronic itch.

Increasing evidence has further demonstrated an important association between gut flora-derived metabolites and the maintenance of cognitive function impairment (19–23). Findings of Yu et al. (20) showed that abnormal gut microbiota composition contributed to the onset of diabetes-induced cognitive dysfunction, suggesting that improving gut microbiota composition may be a potential therapeutic strategy for diabetes and related comorbidities. Yang et al. (21) used hierarchical cluster analysis of sucrose preference test to classify the spared nerve injury (SNI) model rats with or without anhedonia-like phenotype and found that gut microbiota plays a key role in pain as well as depression-like phenotypes including anhedonia in rodents with neuropathic pain. Nevertheless, whether chronic itch-related cognitive function dysfunction is implicated in gut microbiota remains unclear.

In this study, we first investigated the role of gut microbiota in diphenylcyclopropenone (DCP)-evoked chronic itch in mice. Next, we used hierarchical cluster analysis of novel object recognition test (ORT) results to classify the chronic itch in mice with or without cognitive dysfunction (CD)-like phenotype and 16S ribosomal RNA (rRNA) gene sequencing was used to compare gut bacterial composition between CD (Susceptible) and Non-CD phenotypes (Unsusceptible) in chronic itch mice. Furthermore, we examined the effects of fecal bacteria transplantation from chronic itch-induced CD and Non-CD phenotypes on novel object recognition and scratching behaviors of host pseudo-germ-free mice.

## Materials and Methods

### Animals

Male C57/BL6 mice aged 8–10 weeks were supplied by the Experimental Animal Research Center of Hubei Province (Hubei, China). All animals were adapted to their environment 7 days before the experiment. Animals were humanely treated according to the National Institutes of Health Guide for the Care and Use of Laboratory Animals (revised 2011) and the Guide for the Care and Use of Laboratory Animals (National Academic Press, USA; revised 2011). Animals were housed in a temperature-controlled holding room (22 ± 1°C) on a 12-h light/dark cycle and given food and water *ad libitum*. Experimental protocols were approved by the Institutional Animal Care and Use Committee of Tongji Hospital, Tongji Medical College, Huazhong University of Science and Technology, Wuhan, China (IRB ID:TJ-A0803).

### Experimental Design

**Experiment A:** Mice were randomly assigned to two groups: (1) control group (n = 6); (2) DCP group (*n* = 14). Pruritic behaviors were video-recorded and 16S rRNA sequencing was used to analyze the change of gut microbiota.

**Experiment B:** After hierarchical cluster analysis of novel object recognition test was used, mice were divided into the control group (control, *n* = 6), DCP-CD phenotypes (Susceptible, *n* = 6) and DCP-Non-CD phenotypes (Unsusceptible, *n* = 8). Pruritic behaviors were measured and fecal samples were collected for 16S rRNA gene sequencing.

**Experiment C:** Mice were assigned to one of four groups: (1) control group (*n* = 6), mice were given food and water *ad libitum*; (2) PBS group (*n* = 5); (3) Susceptible group (*n* = 7); (4) Unsusceptible group (*n* = 7). PBS (PBS group), fecal suspension of DCP-CD (susceptible group) and DCP-Non-CD (unsusceptible group) were transplanted into pseudo-germ-free mice intragastrically for 14 consecutive days. A novel object recognition test was performed.

### DCP-Evoked Chronic Itch

Mice were shaved on the back of the neck and divided into the DCP group (*n* = 14) and control group (*n* = 6). 0.1 ml 1% DCP (Shanghai Aladdin Biochem Technology Co., Ltd.) dissolved in acetone was painted on the neck skin of mice in the DCP group on day 1 and day 7 under conventional conditions. The neck skin of mice in the control group was painted with 0.1 ml acetone (24). Scratching behaviors were video-recorded on the day before the DCP treatment and day 9 following DCP application.

### Scratching Behaviors in Mice

Mice were habituated in a plastic chamber (9 × 9 × 13 cm) for 15 min before the experiment. The scratching behaviors were video-recorded through a transparent glass under the plastic chamber in the absence of any observer for 30 min. According to our previous research (5, 6, 9, 25, 26), a scratching bout is defined as lifting a hind paw toward the shaved region and returning the hind paw back to the floor or mouth for licking (27). Analyses of the videotapes were carried out in a blinded manner.

### Novel Object Recognition Test

In an open field, two identical objects were placed at two corners 6 cm from each border as previously described (28–30). During the first stage, the animal was allowed free exploration for 5 min and the exploration time around each object was recorded. The next day, the experiment was the same as before except that one of the two objects was replaced by a novel object same in size but different in appearance. The exploration time around the novel object was recorded. The apparatus was wiped with 75% ethanol to eliminate odor after each experiment.

### Feces Sample Collection

Mice were placed in a clean cage with sterile paper on the bottom. The feces were immediately collected in a sterilized centrifuge tube after mice defecated. Fecal samples were stored in −80°C freezer till 16S rRNA gene sequencing analysis. 1 g fecal samples obtained from DCP-CD or DCP-Non-CD mice were diluted in 10 mL aseptic PBS to prepare for fecal transplantation.

### 16S rRNA Gene Sequencing for Fecal Sampling

Based on a previous report (20, 21, 31–33), 16S rRNA gene sequencing was used to perform bioinformatics analysis. Detailed methods are provided in Supplementary Information.

### Pseudo-Germ-Free Mice Modeling and Fecal Transplantation

The pseudo-germ-free mice modeling was prepared as previously reported (20, 21, 34). Briefly, C57BL/6 mice drunk special water containing broad-spectrum antibiotics (ampicillin 1 g/L, neomycin sulfate 1 g/L, metronidazole 1 g/L, Sigma-Aldrich Co. Ltd, USA) *ad libitum* for 14 consecutive days. The drinking solutions were renewed every 2 days.

The fecal material was suspended and each recipient pseudo-germ-free mouse was intragastrically infused with 0.2 mL suspension for 14 consecutive days.

### Statistical Analysis

All quantification data are expressed as means ± SEM, and error bars represent SEM. Statistical analyzes were performed using SPSS software version 17.0 (SPSS Inc., Armonk, New York, USA) and GraphPad Prism software (GraphPad Software, Inc.). Z scores were standardized in Hierarchical cluster analysis. Then, by using the Ward method and Euclidean distance square as distance measurement, ORT results were Hierarchically clustered, and mice were divided into two groups: DCP-CD (susceptible) and DCP-Non-CD (unsusceptible) mice. Behavioral tests were analyzed by one-way or two-way analysis of variance (ANOVA), followed by post hoc Bonferroni's test. *P* < 0.05 was considered to indicate a statistically significant difference.

## Results

### DCP-Evoked Scratching Behaviors in Mice

**Figure 1:**
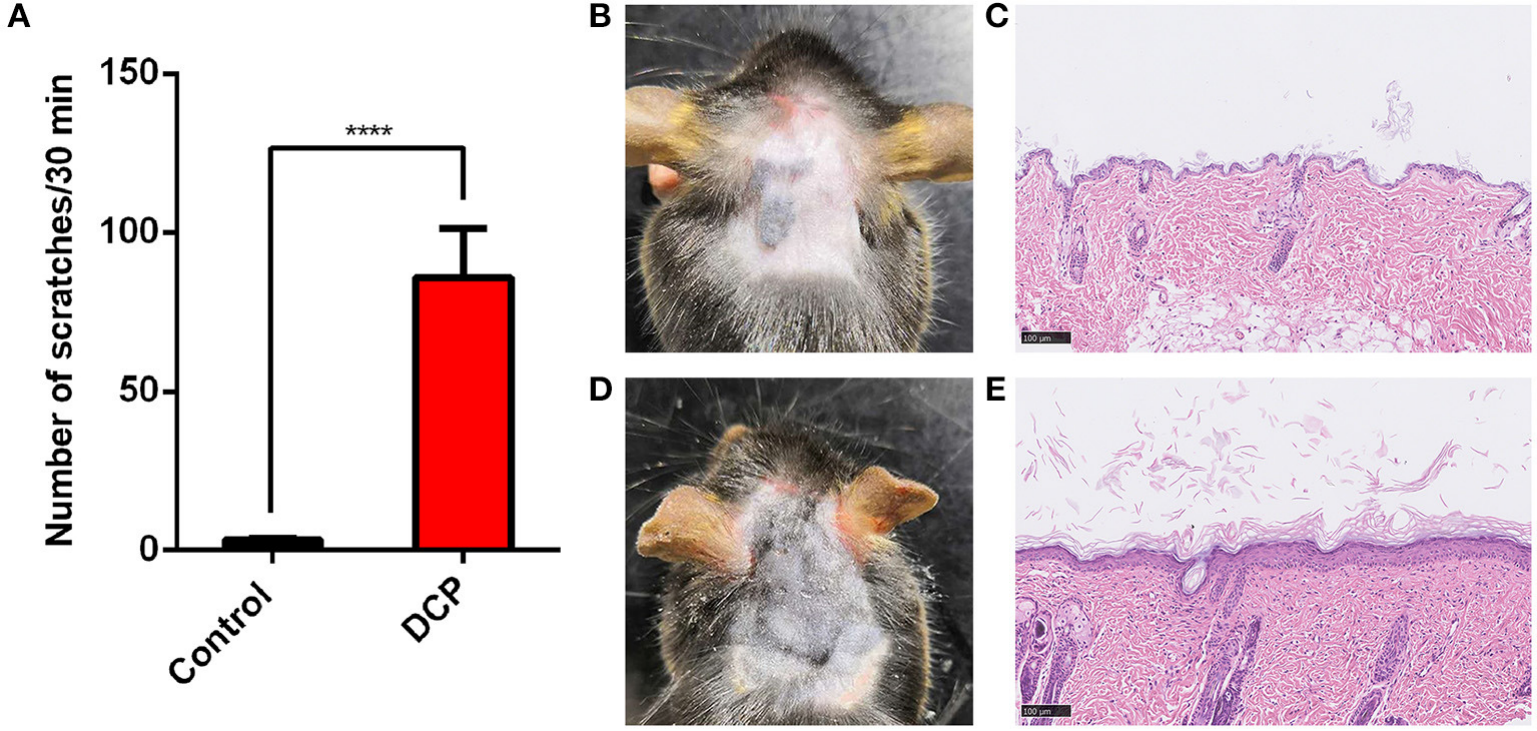
Scratching behaviors and Hematoxylin and eosin (H&E) staining of the neck skin in the control and DCP group. 0.1 ml 1% DCP dissolved in acetone or acetone was painted on the neck skin of the mice on day 1 and day 7 under conventional conditions. Scratching behaviors **(A)** were observed on day 9. Each value represents the mean ± SEM (*n* = 6–14 mice per group). H&E-stained sections from the neck skins treated with **(B,C)** acetone, **(D,E)** DCP. **(B)** A representative image of the neck skin from an acetone treated mouse. The skin was smooth and soft. **(D)** A representative image of the DCP application site from the DCP group. The skin manifested as rough and sclerotic, accompanied by scratches and scabs. ****P < 0.0001 vs. the mice treated with acetone. DCP, diphenylcyclopropenone.

Mice assigned to the DCP group showed a greater number of spontaneous scratching bouts (SSBs) compared to the control group within 30 min of observation. As depicted in Figure 1A, the number of SSBs in the DCP-treated mice (SSB = 85.77 ± 15.56, *n* = 14) showed a significant distinction between the acetone-treated mice at day 10 (SSB = 3.33 ± 0.42, *n* = 6, *P* < 0.0001). Figures 1C,E shows the H&E staining of the skin in the control group and the DCP group. The skin of the control group was smooth and soft while the skin of mice in the DCP group (Figure 1D) was rough and sclerotic, accompanied by scratches and scabs. Compared with the control group (Figure 1B), there was significant epidermal hyperplasia in the DCP group.

### Comparisons of Differential Profiles in Gut Microbiota Among Control, Susceptible and Unsusceptible Mice

**Figure 2:**
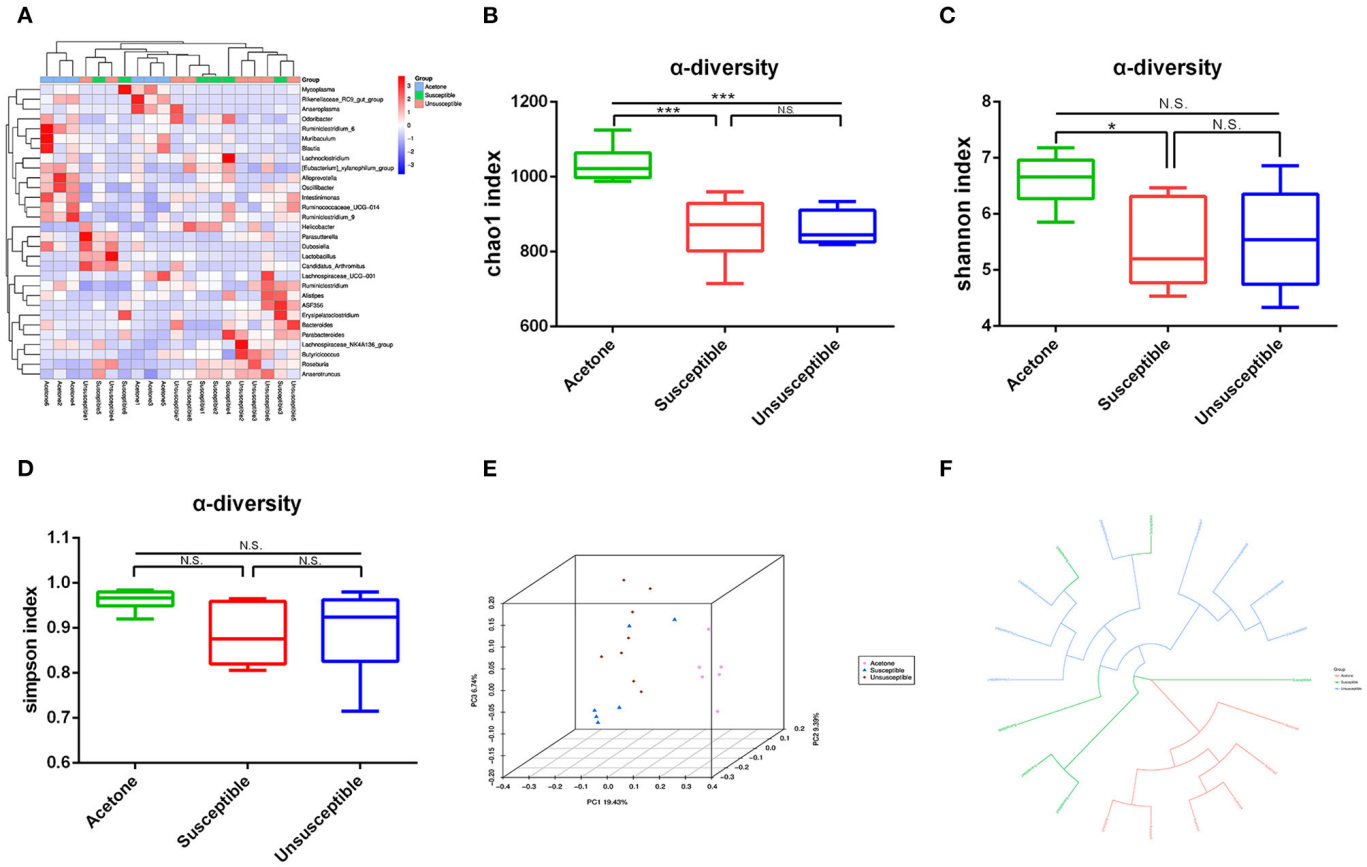
Differential profiles of the gut microbiota among control (acetone), DCP-CD (Susceptible) and DCP-Non-CD (Unsusceptible) mice. **(A)** Heat map of differential levels of bacteria among three groups. **(B)** Chao 1 index between control and susceptible mice (*t*-test, *P* < 0.0001). **(C)** Simpson index between control and susceptible mice (*t*-test, *P* < 0.01). **(D)** Shannon index (*t*-test, *P* < 0.05). **(E)** Chao 1 index. **(F)** Simpson index. PCoA, principal coordinates analysis. *P < 0.05, ***P < 0.0001.

We used 16S rRNA gene sequencing to compare differential profiles of gut microbiota in the two groups. A large number of gut bacteria were altered in three groups (Figure 2A). DCP mice showed a significant decrease in α-diversity value compared with control mice (Figures 2B–D). As depicted in the three-dimensional PCoA picture (Figure 2E), the dots of the DCP group were far apart from the control group. Figure 2F displays the circular tree data, which suggests that the composition of gut microbiota was pretty distinct between the two groups.

### Alterations in the Gut Microbiota Composition Between the DCP and Control Mice

**Figure 3:**
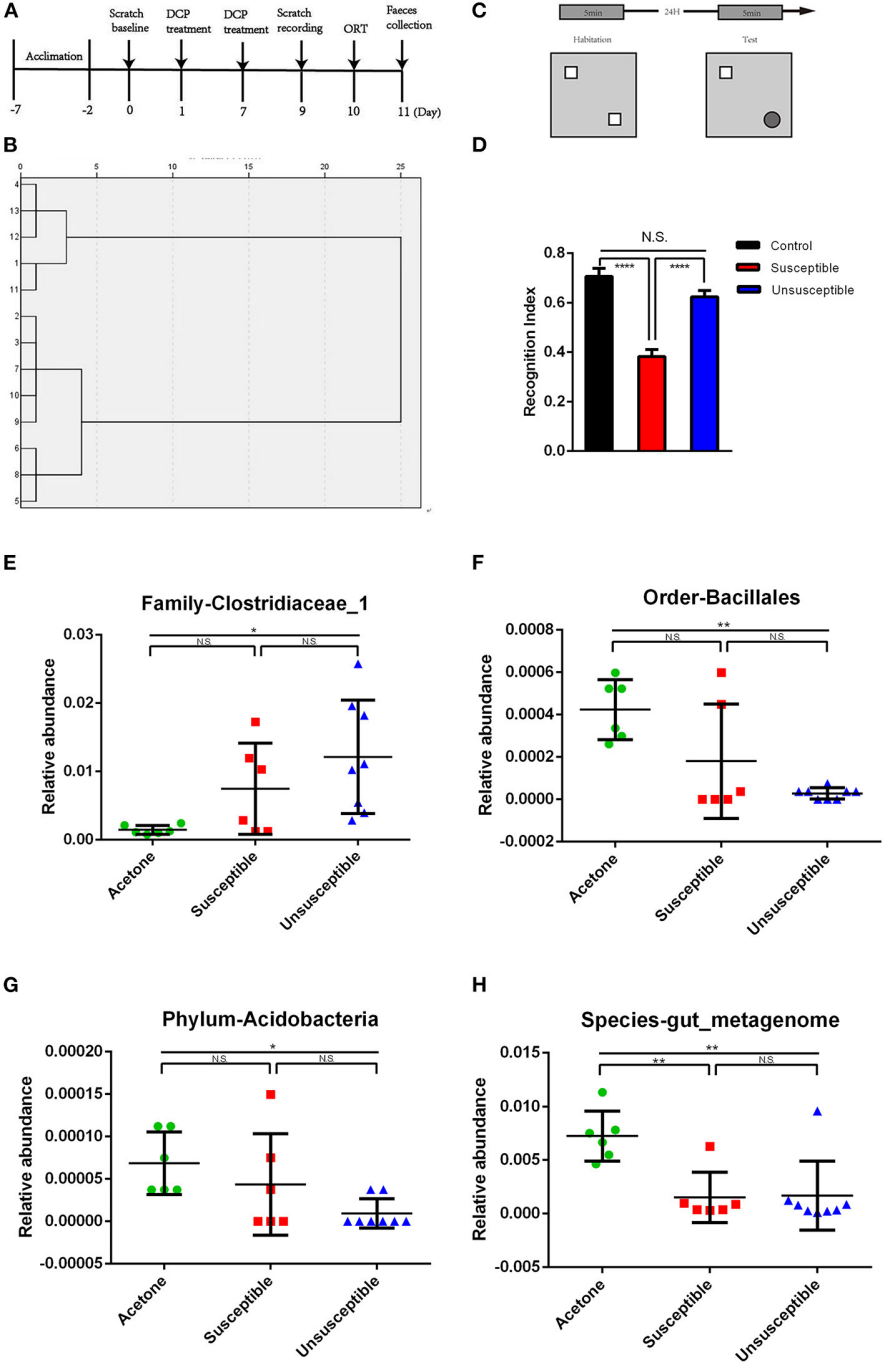
Differences of relative abundance in the gut microbiota among control (acetone), DCP-CD (Susceptible) and DCP-Non-CD (Unsusceptible) mice. **(A)** Timeline of the chronic itch model and behavior tests. Administration of DCP started on day 1 and day 7 after acclimation. Scratching behavior was recorded on day 9. Object recognition test was performed on day 10. Feces was collected on day 11. **(B)** Dendrogram of hierarchical clustering analysis. 14 DCP mice were divided into cognitive dysfunction susceptible and unsusceptible groups by ORT results of hierarchical clustering analysis. **(C)** The schematic presentation for the protocol of novel-object recognition test. **(D)** ORT recognition index [*F*_(2, 16)_ = 29.30, *P* < 0.0001]. **(E)** Family-Clostridiaceae_1 between control and susceptible mice. **(F)** Order-Bacillales. **(G)** Phylum-Acidobacteria. **(H)** Species-gut_metagenome. N.S., not significant; DCP, diphenylcyclopropenone; ORT, Object recognition test. *P < 0.05, **P < 0.001, ****P < 0.0001.

The results of 16S rRNA gene sequencing demonstrate that the alterations of the gut microbiota composition between the DCP and control mice were distinct (Figures 3E–H and Supplementary Figures 1–3). The analysis depicts that 52 bacteria differed between the fecal samples of the DCP and control mice. The relative abundance of 48 bacteria at six phylogenetic levels (phylum, class, order, etc.) was significantly decreased in the DCP mice compared with control mice. On the contrary, the relative abundance of 4 bacteria at genus, phylum and species level increased in the DCP mice compared with control mice. The heat maps of the gut microbiota composition at 6 phylogenetic levels (phylum, class, order, family, genus, and species) outlined specific differences between the control and DCP groups (Supplementary Figures 4, 5).

### Comparisons of Gut Microbiota Among the Control, DCP-CD and DCP-Non-CD Mice

Timeline of the chronic itch model and behavior tests were showed in Figure 3A. Hierarchical cluster analysis was used to classify the result of the novel object recognition test (Figure 3B). The schematic presentation for the protocol of novel-object recognition test was showed in Figure 3C. There was a significant difference in recognition index between susceptible (DCP-CD) or unsusceptible (DCP-Non-CD) mice (Figure 3D). Based on this result, the DCP mice were divided into either the cognition impairment susceptible (DCP-CD) or unsusceptible (DCP-Non-CD) mice. The Chao1 and Simpson indices indicated no significant difference between DCP-CD (Susceptible) and DCP-NCD (Unsusceptible) mice (Figures 2E,F). Moreover, the unweighted unifrac PCoA analysis showed that the DCP-CD and DCP-Non-CD mice might have similar gut microbiota composition (Figure 2G). A Binary Jaccard circular tree suggested there were similarities between the DCP-CD and DCP-Non-CD mice (Figure 2H).

### Effects of Fecal Microbiota Transplantation From DCP-CD or DCP-Non-CD Mice on Short-Term Memory in Pseudo-Germ-Free Mice

**Figure 4:**
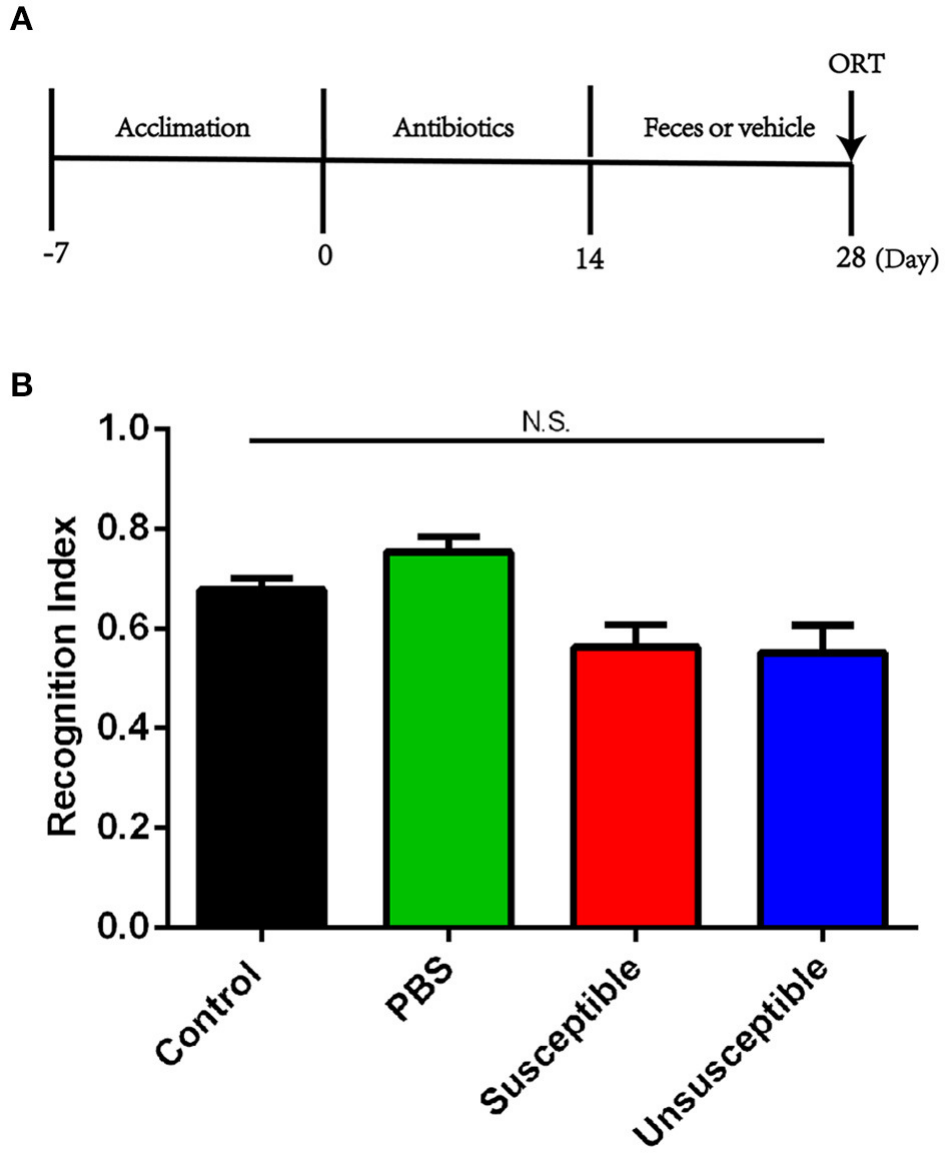
Effects of fecal microbiota transplantation from DCP-CD or DCP-Non-CD mice on cognitive dysfunction in pseudo-germ-free mice. **(A)** Timeline of the establishment of pseud-germ-free mice and fecal transplantation. Antibiotics were dissolved in drinking water for 14 consecutive days. Fecal transplantation was performed for the other 14 consecutive days. ORT was performed on day 28. **(B)** ORT recognition index. N.S., not significant; ORT, Object recognition test.

Pseudo-germ-free mice were established by adding a large dose of antibiotics into the drinking water for 14 consecutive days. For another consecutive 14 days, fecal microbiotas were transplanted on pseudo-germ-free mice by intragastric administration (Figure 4A). Recognition index between mice receiving transplantation of fecal samples from susceptible or unsusceptible mice showed no significant difference (Figure 4B).

## Discussion

This study provides novel insights into the vital role of gut microbiota in the process of chronic itch. Our findings are as follows: (1) There are abnormal compositions of gut microbiota in DCP-evoked chronic itch mice; (2) chronic itch may induce novel object recognition-related CD; (3) There are no differential alterations for abnormal gut microbiota compositions between chronic itch mice with and without CD; (4) Fecal microbiota transplantation from chronic itch mice with or without CD do not induce ORT-related CD in the pseudo-germ-free mice. Chronic itch is a common manifestation of a number of inflammatory skin diseases including contact dermatitis. Clinical studies have reported that DCP, an immunotherapy agent for patients with alopecia areata, induces eczematous skin diseases including contact dermatitis and severe pruritus. Contact dermatitis evoked by DCP has become a model system for studying chronic itch (27, 35). We successfully established DCP-evoked chronic itch model in C57BL/6J mice and found persistent scratching behavior and skin histopathological data. These results are in line with previous reports (9, 36). A vast array of clinical data show that chronic itch can cause the alteration of cognitive schemas (37, 38), for example, more negative memories and expectations with regard to itch. Our behavior evidences in mice strongly support that DCP-induced chronic itch can lead to novel object recognition-related cognitive dysfunction.

It is well-known that the impact of the wide variety of intestinal microbiota on numerous functions of the central nervous system has been increasingly recognized (39, 40). After the spared nerve injury (SNI) model rats were divided into SNI-CD phenotypes (Susceptible) and SNI-Non-CD phenotypes (Unsusceptible), Yang et al. (21) showed that abnormal gut microbiota composition induced by SNI correlated with anhedonia-related CD, suggesting that gut microbiota plays a key role in the neuropathic pain with depression-like phenotypes including anhedonia. Therefore, we speculate that chronic itch-related cognitive dysfunction may be implicated in gut microbiota. The 16S rRNA gene sequencing technology is a unique and powerful tool for revealing the gut microbiota composition and relationships between physiological functions and pathological features to date (41–44). α-diversity is defined as the degree of the species diversity in a biological environment and is mainly concerned with the number of bacteria or species therein (45). The Chao 1, Shannon, and Simpson indices are commonly used to evaluate the α-diversity of microbiota. In the present study, all three indices showed a significant decrease in fecal samples from the DCP mice than from control mice. PcoA demonstrated the differences between individuals or groups. The closer the distance is, the greater the biological repetition is within the same group. The distance of different groups represents their similarity or difference. As depicted in the three-dimensional PCoA picture, the dots of the DCP group were far apart from the control group. Circular tree data also illustrates that the composition of gut microbiota was pretty distinct between the two groups.

In our previous research, using diphenylcyclopropenone (DCP)- and acetone/ether/water (AEW)-induced chronic itch models, we showed that chronic itch did (not DCP or AEW) result in the changes of multiple mediators, such as chemokines in the spinal cord (9). In our other study, we demonstrated that alpha-Me-5-HT- and histamine-evoked acute pruritus induced specific patterns of spinal metabolites assessed by proton nuclear magnetic resonance spectroscopy, not alpha-Me-5-HT or histamine induced specific patterns of spinal metabolites (5). Thereby, we think that the alteration of gut microbiota is due to DCP-induced chronic itch, but not DCP treatment *per se* (Reviewer's suggestion). We used this method to reveal that 52 bacteria were altered at 6 levels in the DCP group compared to the control mice. At the genus level, *uncultured_bacterium, Mycoplasma, Alloprevotella, Rikenellaceae_RC9_gut_group, Anaeroplasma, Muribaculum, Ruminiclostridium_6, Blautia, uncultured_organism, Prevotel-laceae_UCG-001, Ruminococcus_1, Ruminiclostridium_5, Prevotella, Azospirillum_sp._47_25, Dialister* were significantly decreased in DCP mice compared with control group. On the contrary, *Helicobacter, Roseburia, Anaerotruncus, ASF356, Candidatus_Arthromitus, Erysipelato-clostridium, Clostridium_sensu_stricto_1, GCA-900066225, Negativibacillus, Lactococcs, Ru-minococcaceae_UCG-004, Romboutsia, [Clostridium]_innocuum_group, Prevotella_9, Fusobact-erium* were significantly increased in DCP mice. These results suggest that gut microbiota may play an important role in DCP-evoked chronic itch in mice. At the species level, *uncultured_bacterium, Lachnospiraceae_bacterium_28-4, gut_metagenome, Lachnospiraceae_bacterium_COE1, Bacteroides_fragilis, Azospirillum_sp._47_25, uncultured_Ali-stipes_sp*. were significantly decreased in the DCP mice compared with the control group. In contrast, *uncultured_Clostridiales_bacterium, Lactobacillus_murinus, Clostridium_butyricum, Clostri-dium_sp._ND2, Lactococcus_lactis* were significantly increased in DCP mice. It seems that abnormal composition of these microbiota may play a role in DCP-evoked chronic itch.

It has been reported that the alterations in the composition of gut microbes may effect cognitive function (46–49). We adopted hierarchical cluster analysis of novel object recognition performance indices to classify the chronic itch model in mice into the DCP-CD (Susceptible) and DCP-Non-CD (Unsusceptible) phenotypes. In the present study, we observed no significant difference in α-diversity (consisting of Shannon and Simpson indices) among the Susceptible and Unsusceptible groups, suggesting little change in bacterial numbers in the two groups. In addition, the separation of groups according to β-diversity (PCoA) indicates that the microbiota composition was not significantly altered by the novel object recognition-related CD. These results are consistent with our findings that gut bacteria were not significantly different in the fecal samples between Control, Susceptible and Unsusceptible groups using 16S rRNA gene sequencing (Supplementary Figure 6). We, therefore, propose that gut bacteria might be not correlated with novel object recognition-related CD in the chronic itch mice.

Using large doses of antibiotics to establish pseudo-germ-free mice has become a common approach of fecal microbiota transplant studies (50, 51). In the present study, we observed that fecal microbiota transplant from DCP-CD (Susceptible) and DCP-Non-CD (Unsusceptible) mice did not induce novel object recognition-related CD, supporting the notion that regulating gut microbiota composition cannot improve chronic itch-induced CD.

In conclusion, these findings suggest that the microbiota composition was significantly altered by DCP-evoked chronic itch and that chronic itch may induce novel object recognition-related CD. However, abnormal gut microbiota composition induced by chronic itch may not be correlated with novel object recognition-related CD.

## Data Availability Statement

The datasets presented in this study can be found in online repositories. The names of the repository/repositories and accession number(s) can be found in the article/Supplementary Material.

## Ethics Statement

The animal study was reviewed and approved by Experimental protocols were approved by Institutional Animal Care and Use Committee of Tongji Hospital, Tongji Medical College, Huazhong University of Science and Technology, Wuhan, China (IRB ID:TJ-A0803).

## Author Contributions

All authors listed have made a substantial, direct and intellectual contribution to the work, and approved it for publication.

## Conflict of Interest

The authors declare that this study received assistance from OEbiotech Co. Ltd. The affiliation was not involved in the study design, collection, analysis, interpretation of data, the writing of this article or the decision to submit it for publication.

## Supplementary Material

Supplementary Information 16S rRNA analysis of fecal samples: The 16S rRNA analysis of fecal samples was performed at OEbiotech Co. Ltd. (Shanghai, China). DNA extraction was performed using DNA Extraction Kit following the manufacturer's instructions. V3-V4 (or V4-V5) variable regions of 16S rRNA genes were amplified with universal primers 343 F and 798 R. PCR products were then purified for further sequencing. Clean reads were subjected to primer sequences removal and clustering to generate operational taxonomic units (OTUs) using Vsearch software with 97% similarity cutoff (27). All representative reads were annotated and blasted against Silva database Version 123 (or Greengens) using RDP classifier (confidence threshold was 70%) (32). All representative reads were annotated and blasted against Unite database (ITSs rDNA) using blast (33).

Supplementary Figure 1 Differences in the relative abundance of various gut microbes between control and DCP mice. **(A)** Cla2ss-Mollicutes (*t*-test. *P* = 0.0117). **(B)** Class-Alphaproteobacteria (*t*-test, *P* = 0.0109). **(C)** Class-Other (*t*-test. *P* = 0.0297). **(D)** Class-Fus-obacteriia (*t*-test. *P* = 0.0029). **(E)** Class-Negativicutes (*t*-test, *P* = 0.0401). **(F)** Class-Melainabacteria (*t*-test, *P* = 0.0051). **(G)** Class-Thermoanaerobaculia (*t*-test, *P* = 0.0089). **(H)** Class-Gemmatimona-detes (*t*-test, *P* = 0.0139). **(I)** Family-Prevotellaceae (*t*-test, *P* = 0.0244). **(J)** Family-Prevotellaceae (*t*-test, *P* = 0.0064). **(K)** Family-Other (*t*-test, *P* < 0.0001). **(L)** Family-Flavobacteriaceae (*t*-test, *P* = 0.0031). **(M)** Family-Fusobacteriaceae (*t*-test, *P* = 0.0017). **(N)** Family-Staphylococcaceae (*t*-test, *P* = 0.0011). **(O)** Genus-Mycoplasma (*t*-test, *P* = 0.0061). **(P)** Genus-Alloprevotella (*t*-test, *P* = 0.0326). **(Q)** Genus-Roseburia (*t*-test, *P* = 0.0021). **(R)** Genus-Anaerotruncus (*t*-test, *P* = 0.0086). **(S)** Genus-Riken-ellaceae_RC9_gut_group (*t*-test, *P* < 0.0001). **(T)** Genus-ASF356 (*t*-test, *P* = 0.0341).

Supplementary Figure 2 Differences in the relative abundance of various gut microbes between control and DCP mice. **(A)** Genus-Anaeroplasma (*t*-test, *P* = 0.0304). **(B)** Genus-Ruminiclostridium_6 (*t*-test, *P* = 0.0002). **(C)** Genus-Blautia (*t*-test, *P* = 0.0256). **(D)** Genus-uncultured_organism (*t*-test, *P* = 0.0198). **(E)** Genus-Prevotellaceae_UCG-001 (*t*-test, *P* = 0.0209). **(F)** Genus-Ruminococcus_1 (*t*-test, *P* < 0.0001). **(G)** Genus-GCA-900066225 (*t*-test, *P* = 0.0213). **(H)** Genus-Negativibacillus (*t*-test, *P* = 0.0342). **(I)** Genus-Ruminiclostridium_5 (*t*-test, *P* = 0.0425). **(J)** Genus-Ruminococcaceae_UCG-004 (*t*-test, *P* = 0.0478). **(K)** Genus-Lactococcus (*t*-test, *P* < 0.0001). **(L)** Genus-Prevotella (*t*-test, *P* = 0.0006). **(M)** Genus-Romboutsia (*t*-test, *P* = 0.0301). **(N)** Genus-[Clostridium]_innocuum_group (*t*-test, *P* = 0.0158). **(O)** Genus-Prevotella_9 (*t*-test, *P* = 0.0219). **(P)** Genus-Azospirillum_sp._47_25 (*t*-test, *P* = 0.0034). **(Q)** Genus-Dialister (*t*-test, *P* = 0.0401). **(R)** Genus-Fusobacterium (*t*-test, *P* = 0.0017). **(S)** Order-Anaeroplasmatales (*t*-test, *P* = 0.0304). **(T)** Order-Rhodospirillales (*t*-test, *P* = 0.0124).

Supplementary Figure 3 Differences in the relative abundance of various gut microbes between control and DCP mice**. (A)** Order-Other (*t*-test, *P* = 0.0076). **(B)** Order-Fusobacteriales (*t*-test, *P* = 0.0029). **(C)** Order-Gastranaerophilales (*t*-test, *P* = 0.0051). **(D)** Order-Thermoanaerobaculales (*t*-test, *P* = 0.0089). **(E)** Order-Bacillales (*t*-test, *P* = 0.0027). **(F)** Phylum-Tenericutes (*t*-test, *P* = 0.0117). **(G)** Phylum-Other (*P* = 0.0142). **(H)** Phylum-Fusobacteria (*t*-test, *P* = 0.0029). **(I)** Phylum-Cyanobacteria (*t*-test, *P* = 0.0051). **(J)** Phylum-Acidobacteria (*t*-test, *P* = 0.0109). **(K)** Phylum-Gemmatimonadetes (*t*-test, *P* = 0.0139). **(L)** Species-uncultured_Clostridiales_bacterium (*t*-test, *P* = 0.0227). **(M)** Species-Lachnospiraceae_bacterium_28-4 (*t*-test, *P* = 0.0092). **(N)** Species-Lachnospiraceae_bacterium_COE1 (*t*-test, *P* = 0.0022). **(O)** Species-Bacteroides_fragilis (*t*-test, *P* = 0.0272). **(P)** Species-Clostridium_butyricum (*t*-test, *P* = 0.0018). **(Q)** Species-Clostridium_sp._ND2 (*t*-test, *P* = 0.0141). **(R)** Species-Azospirillum_sp._47_25 (*t*-test, *P* = 0.0034). **(S)** Species-uncultured_Alistipes_sp (*t*-test, *P* = 0.0009). **(T)** Species-Lac-tococcus_lactis (*t*-test, *P* = 0.0393).

Supplementary Figure 4 Heatmaps of gut microbiota composition at class, family, and genus levels for control and DCP mice. **(A)** Heatmap (class level). **(B)** Heatmap (family level). **(C)** Heatmap (genus level).

Supplementary Figure 5 Heatmaps of gut microbiota composition at order, phylum, and species levels for control and DCP mice. **(A)** Heatmap (order level). **(B)** Heatmap (phylum level). **(C)** Heatmap (species level).

Supplementary Figure 6 Differences in relative abundance of gut microbiota at various phylogenetic levels among control (acetone), DCP-CD (Susceptible) and DCP-Non-CD (Unsusceptible) mice. **(A)** Class-Bacteroidia (one-way ANOVA, *F*_(2, 17)_ = 7.894, *P* = 0.0038). **(B)** Class-Fusobacteriia (one-way ANOVA, *F*_(2, 17)_ = 8.708, *P* = 0.0025). **(C)** Family-Muribaculaceae (one-way ANOVA, *F*_(2, 17)_ = 9.514, *P* = 0.0017). **(D)** Family-Prevotellaceae (one-way ANOVA, *F*_(2, 17)_ = 4.296, *P* = 0.0309). **(E)** Family-Rikenellaceae (one-way ANOVA, *F*_(2, 17)_ = 5.805, *P* = 0.0120). **(F)** Family-Clostridiaceae_1 (one-way ANOVA, *F*_(2, 17)_ = 4.723, *P* = 0.0234). **(G)** Genus-Blautia (one-way ANOVA, *F*_(2, 17)_ = 5.117, *P* = 0.0182). **(H)** Genus-uncultured_organism (one-way ANOVA, *F*_(2, 17)_ = 5.396, *P* = 0.0153). **(I)** Genus-uncultured_organism (one-way ANOVA, *F*_(2, 17)_ = 4.168, *P* = 0.0336). **(J)** Genus-Clostridium_sensu_stricto_1 (one-way ANOVA, *F*_(2, 17)_ = 4.168, *P* = 0.0336). **(K)** Genus-Prevotella (one-way ANOVA, *F*_(2, 17)_ = 14.52, *P* = 0.0002). **(L)** Genus-Az-ospirillum_sp._47_25 (one-way ANOVA, *F*_(2, 17)_ = 4.208, *P* = 0.0328). **(M)** Order-Bacteroidales (one-way ANOVA, *F*_(2, 17)_ = 7.623, *P* = 0.0043). **(N)** Order-Flavobacteriales (one-way ANOVA, *F*_(2, 17)_ = 8.464, *P* = 0.0028). **(O)** Order-Fusobacteriales (one-way ANOVA, *F*_(2, 17)_ = 8.708, *P* = 0.0025). **(P)** Phylum-B-acteroidetes (one-way ANOVA, *F*_(2, 17)_ = 7.894, *P* = 0.0038). **(Q)** Phylum-Bacteroidetes (one-way A-NOVA, *F*_(2, 17)_ = 4.180, *P* = 0.0334). **(R)** Phylum-Fusobacteria (one-way ANOVA, *F*_(2, 17)_ = 8.708, *P* = 0.0025). **(S)** Species-Lactobacillus_murinus (one-way ANOVA, *F*_(2, 17)_ = 4.184, *P* = 0.0333). **(T)** Species-gut_metagenome (one-way ANOVA, *F*_(2, 17)_ = 8.906, *P* = 0.0023).
