# Supplementary figures and images for: The Role of Gut Microbiota in Chronic Itch-Evoked Novel Object Recognition-Related Cognitive Dysfunction in Mice
Source: Front Med (Lausanne). 2021 Feb 5;8:616489. doi: 10.3389/fmed.2021.616489 (PMC7892771; doi:10.3389/fmed.2021.616489)

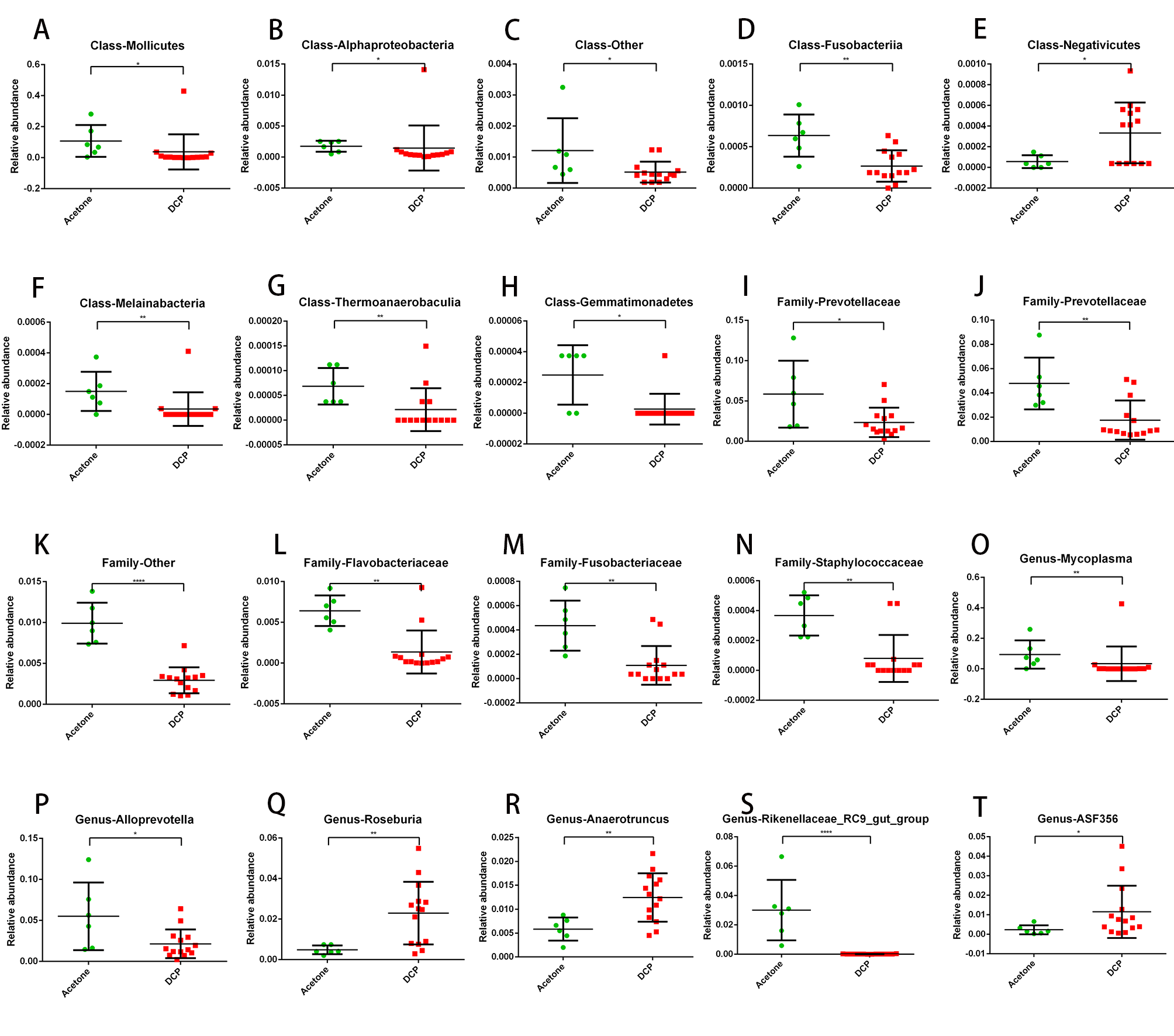

Supplement: Supplementary Figure 1 — Differences in the relative abundance of various gut microbes between control and DCP mice. (A) Cla2ss-Mollicutes (t-test. P = 0.0117). (B) Class-Alphaproteobacteria (t-test, P = 0.0109). (C) Class-Other (t-test. P = 0.0297). (D) Class-Fus-obacteriia (t-test. P = 0.0029). (E) Class-Negativicutes (t-test, P = 0.0401). (F) Class-Melainabacteria (t-test, P = 0.0051). (G) Class-Thermoanaerobaculia (t-test, P = 0.0089). (H) Class-Gemmatimona-detes (t-test, P = 0.0139). (I) Family-Prevotellaceae (t-test, P = 0.0244). (J) Family-Prevotellaceae (t-test, P = 0.0064). (K) Family-Other (t-test, P < 0.0001). (L) Family-Flavobacteriaceae (t-test, P = 0.0031). (M) Family-Fusobacteriaceae (t-test, P = 0.0017). (N) Family-Staphylococcaceae (t-test, P = 0.0011). (O) Genus-Mycoplasma (t-test, P = 0.0061). (P) Genus-Alloprevotella (t-test, P = 0.0326). (Q) Genus-Roseburia (t-test, P = 0.0021). (R) Genus-Anaerotruncus (t-test, P = 0.0086). (S) Genus-Riken-ellaceae_RC9_gut_group (t-test, P < 0.0001). (T) Genus-ASF356 (t-test, P = 0.0341). [file Image_1.tif]

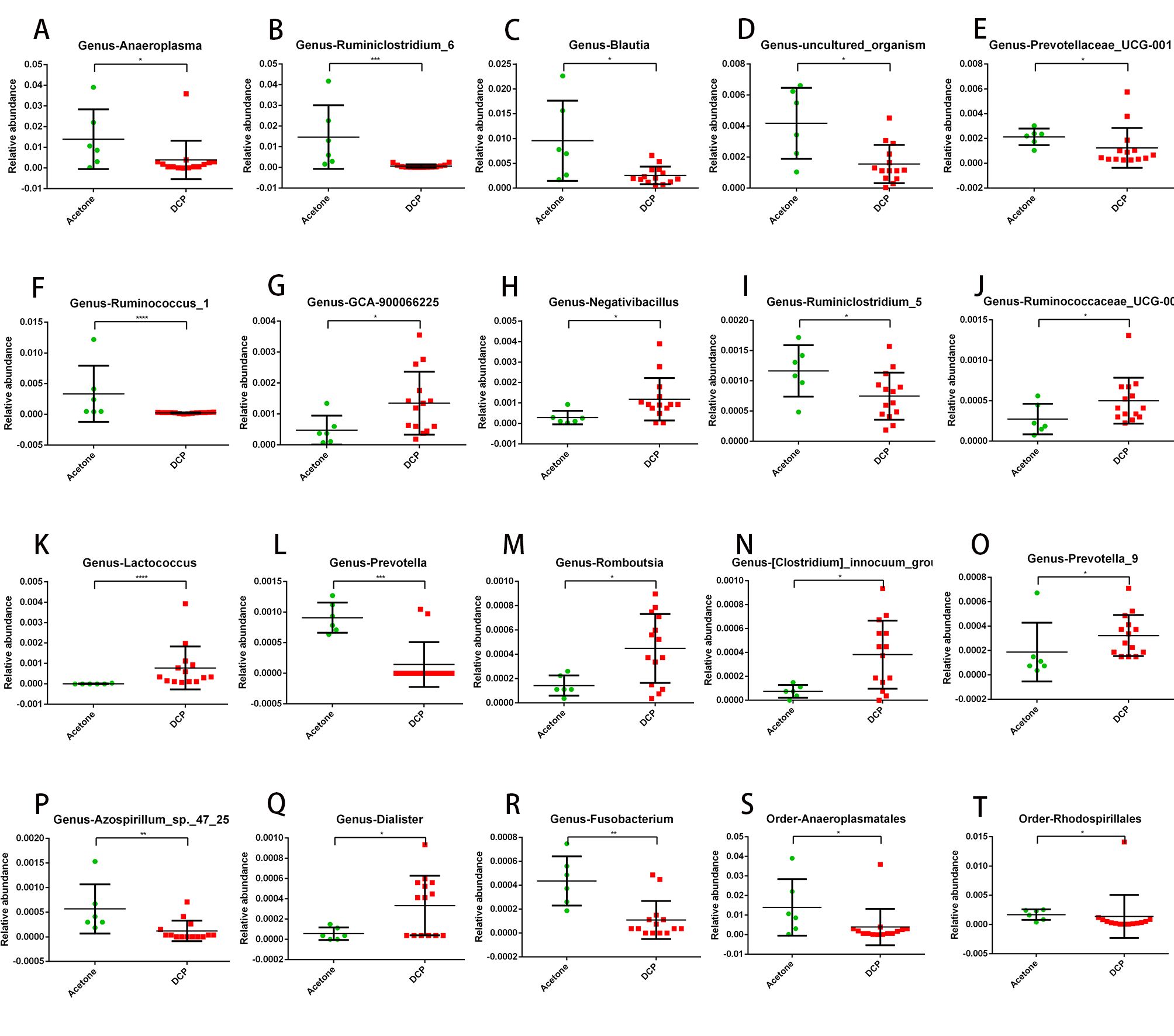

Supplement: Supplementary Figure 2 — Differences in the relative abundance of various gut microbes between control and DCP mice. (A) Genus-Anaeroplasma (t-test, P = 0.0304). (B) Genus-Ruminiclostridium_6 (t-test, P = 0.0002). (C) Genus-Blautia (t-test, P = 0.0256). (D) Genus-uncultured_organism (t-test, P = 0.0198). (E) Genus-Prevotellaceae_UCG-001 (t-test, P = 0.0209). (F) Genus-Ruminococcus_1 (t-test, P < 0.0001). (G) Genus-GCA-900066225 (t-test, P = 0.0213). (H) Genus-Negativibacillus (t-test, P = 0.0342). (I) Genus-Ruminiclostridium_5 (t-test, P = 0.0425). (J) Genus-Ruminococcaceae_UCG-004 (t-test, P = 0.0478). (K) Genus-Lactococcus (t-test, P < 0.0001). (L) Genus-Prevotella (t-test, P = 0.0006). (M) Genus-Romboutsia (t-test, P = 0.0301). (N) Genus-[Clostridium]_innocuum_group (t-test, P = 0.0158). (O) Genus-Prevotella_9 (t-test, P = 0.0219). (P) Genus-Azospirillum_sp._47_25 (t-test, P = 0.0034). (Q) Genus-Dialister (t-test, P = 0.0401). (R) Genus-Fusobacterium (t-test, P = 0.0017). (S) Order-Anaeroplasmatales (t-test, P = 0.0304). (T) Order-Rhodospirillales (t-test, P = 0.0124). [file Image_2.tif]

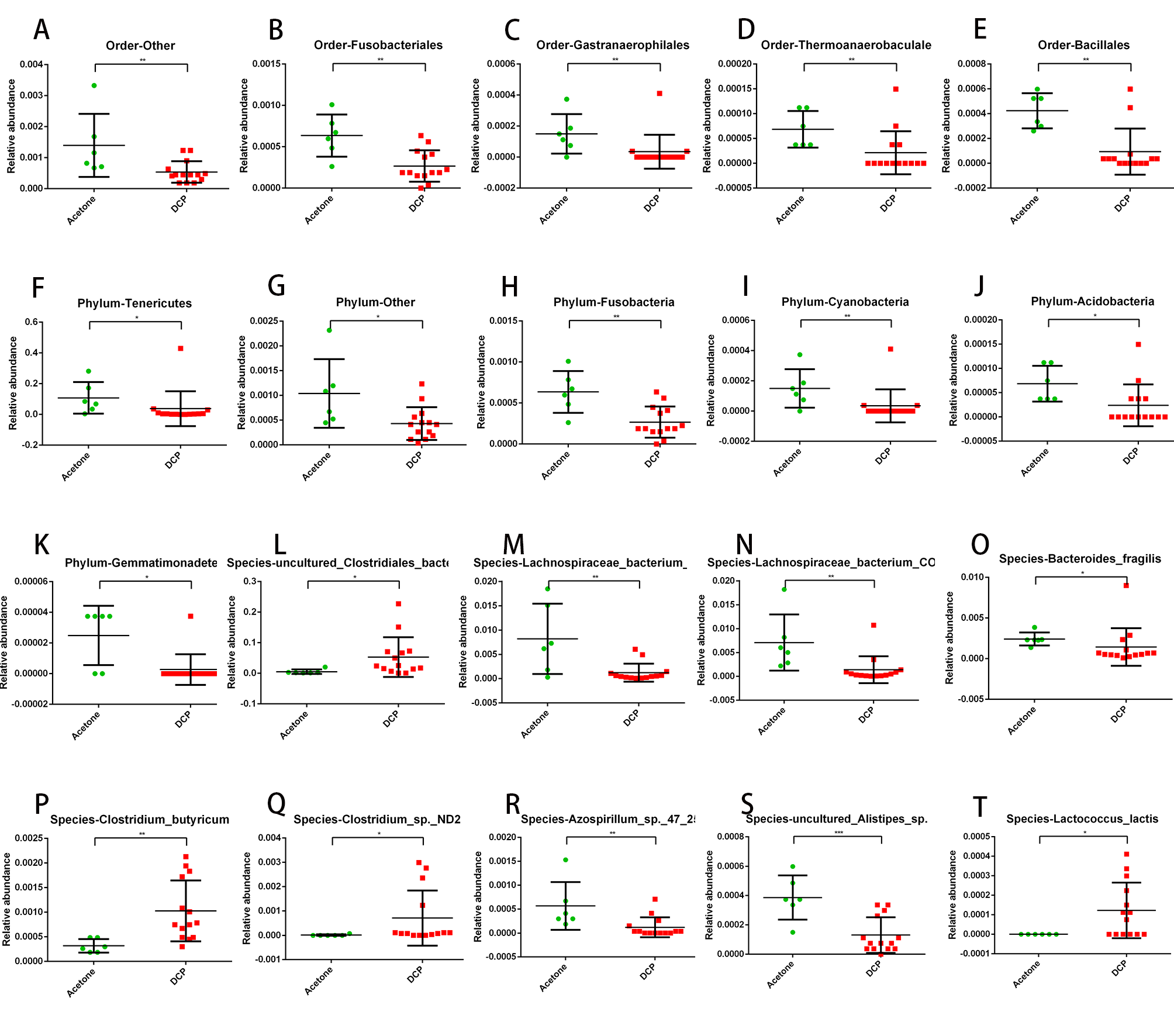

Supplement: Supplementary Figure 3 — Differences in the relative abundance of various gut microbes between control and DCP mice. (A) Order-Other (t-test, P = 0.0076). (B) Order-Fusobacteriales (t-test, P = 0.0029). (C) Order-Gastranaerophilales (t-test, P = 0.0051). (D) Order-Thermoanaerobaculales (t-test, P = 0.0089). (E) Order-Bacillales (t-test, P = 0.0027). (F) Phylum-Tenericutes (t-test, P = 0.0117). (G) Phylum-Other (P = 0.0142). (H) Phylum-Fusobacteria (t-test, P = 0.0029). (I) Phylum-Cyanobacteria (t-test, P = 0.0051). (J) Phylum-Acidobacteria (t-test, P = 0.0109). (K) Phylum-Gemmatimonadetes (t-test, P = 0.0139). (L) Species-uncultured_Clostridiales_bacterium (t-test, P = 0.0227). (M) Species-Lachnospiraceae_bacterium_28-4 (t-test, P = 0.0092). (N) Species-Lachnospiraceae_bacterium_COE1 (t-test, P = 0.0022). (O) Species-Bacteroides_fragilis (t-test, P = 0.0272). (P) Species-Clostridium_butyricum (t-test, P = 0.0018). (Q) Species-Clostridium_sp._ND2 (t-test, P = 0.0141). (R) Species-Azospirillum_sp._47_25 (t-test, P = 0.0034). (S) Species-uncultured_Alistipes_sp (t-test, P = 0.0009). (T) Species-Lac-tococcus_lactis (t-test, P = 0.0393). [file Image_3.tif]

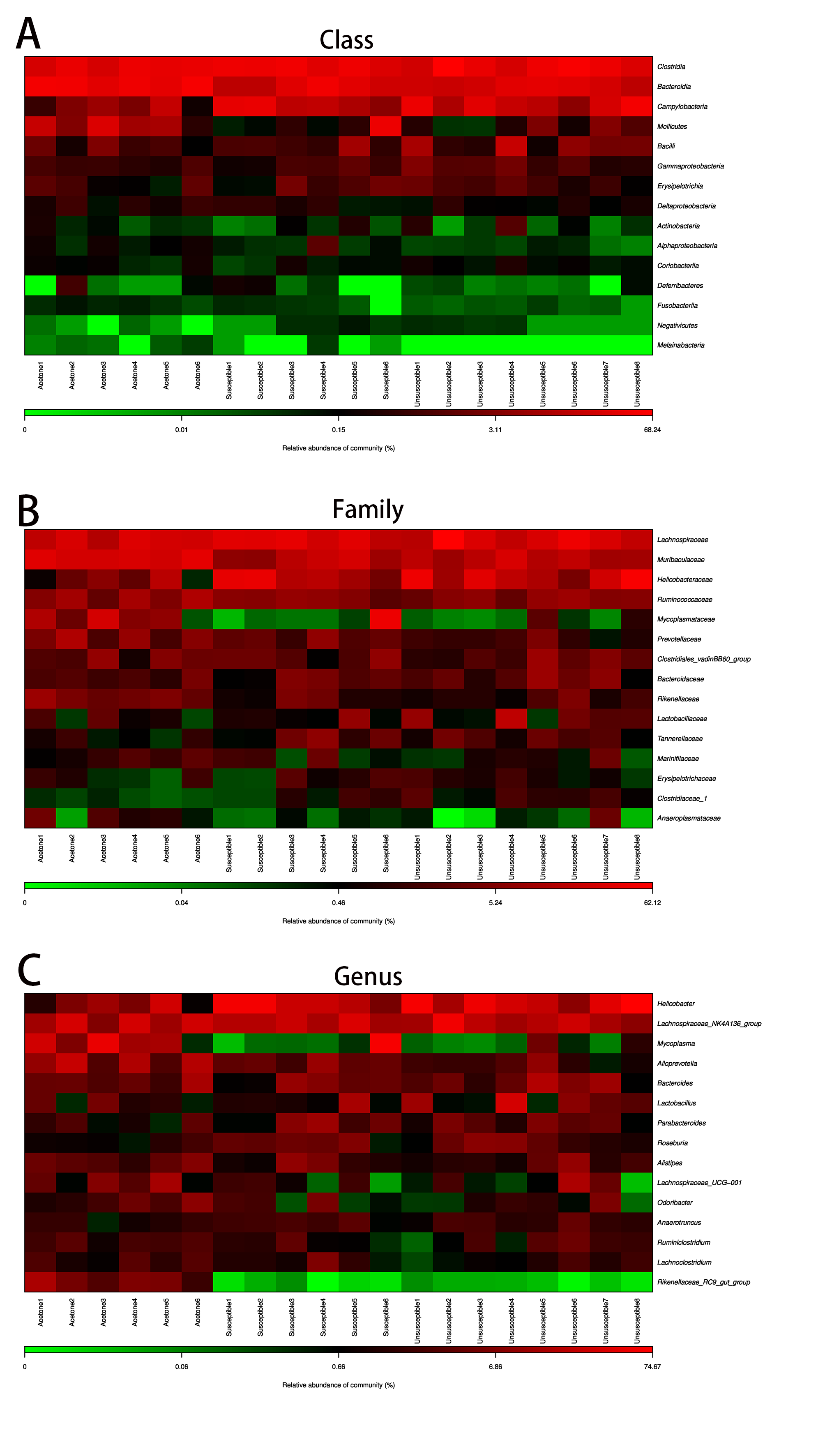

Supplement: Supplementary Figure 4 — Heatmaps of gut microbiota composition at class, family, and genus levels for control and DCP mice. (A) Heatmap (class level). (B) Heatmap (family level). (C) Heatmap (genus level). [file Image_4.tif]

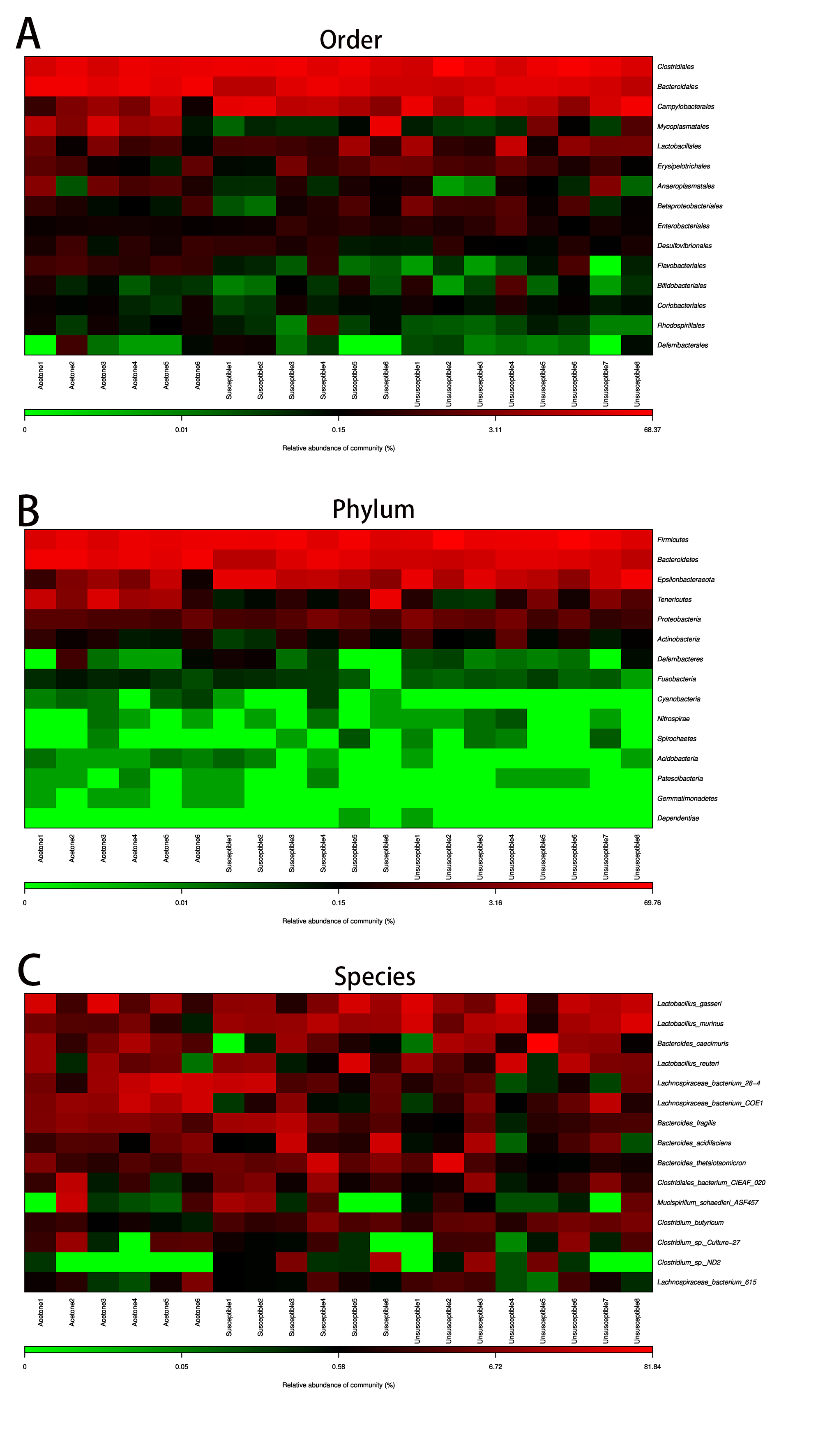

Supplement: Supplementary Figure 5 — Heatmaps of gut microbiota composition at order, phylum, and species levels for control and DCP mice. (A) Heatmap (order level). (B) Heatmap (phylum level). (C) Heatmap (species level). [file Image_5.tif]

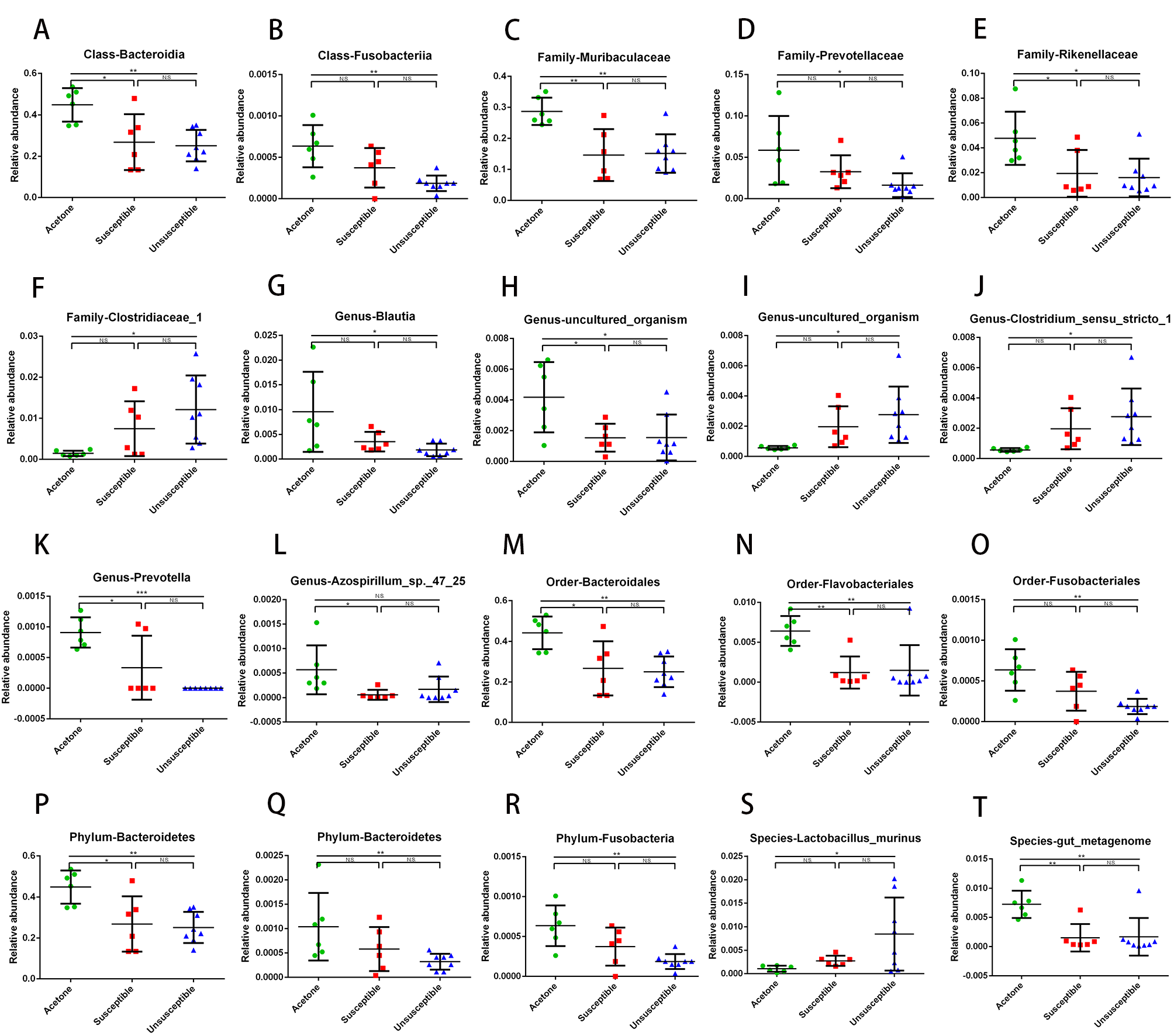

Supplement: Supplementary Figure 6 — Differences in relative abundance of gut microbiota at various phylogenetic levels among control (acetone), DCP-CD (Susceptible) and DCP-Non-CD (Unsusceptible) mice. (A) Class-Bacteroidia (one-way ANOVA, F(2, 17) = 7.894, P = 0.0038). (B) Class-Fusobacteriia (one-way ANOVA, F(2, 17) = 8.708, P = 0.0025). (C) Family-Muribaculaceae (one-way ANOVA, F(2, 17) = 9.514, P = 0.0017). (D) Family-Prevotellaceae (one-way ANOVA, F(2, 17) = 4.296, P = 0.0309). (E) Family-Rikenellaceae (one-way ANOVA, F(2, 17) = 5.805, P = 0.0120). (F) Family-Clostridiaceae_1 (one-way ANOVA, F(2, 17) = 4.723, P = 0.0234). (G) Genus-Blautia (one-way ANOVA, F(2, 17) = 5.117, P = 0.0182). (H) Genus-uncultured_organism (one-way ANOVA, F(2, 17) = 5.396, P = 0.0153). (I) Genus-uncultured_organism (one-way ANOVA, F(2, 17) = 4.168, P = 0.0336). (J) Genus-Clostridium_sensu_stricto_1 (one-way ANOVA, F(2, 17) = 4.168, P = 0.0336). (K) Genus-Prevotella (one-way ANOVA, F(2, 17) = 14.52, P = 0.0002). (L) Genus-Az-ospirillum_sp._47_25 (one-way ANOVA, F(2, 17) = 4.208, P = 0.0328). (M) Order-Bacteroidales (one-way ANOVA, F(2, 17) = 7.623, P = 0.0043). (N) Order-Flavobacteriales (one-way ANOVA, F(2, 17) = 8.464, P = 0.0028). (O) Order-Fusobacteriales (one-way ANOVA, F(2, 17) = 8.708, P = 0.0025). (P) Phylum-B-acteroidetes (one-way ANOVA, F(2, 17) = 7.894, P = 0.0038). (Q) Phylum-Bacteroidetes (one-way A-NOVA, F(2, 17) = 4.180, P = 0.0334). (R) Phylum-Fusobacteria (one-way ANOVA, F(2, 17) = 8.708, P = 0.0025). (S) Species-Lactobacillus_murinus (one-way ANOVA, F(2, 17) = 4.184, P = 0.0333). (T) Species-gut_metagenome (one-way ANOVA, F(2, 17) = 8.906, P = 0.0023). [file Image_6.tif]
